# Comparative Analysis of Teaching at Public Universities in Sinaloa during Confinement Due to COVID-19

**DOI:** 10.3390/ijerph19137687

**Published:** 2022-06-23

**Authors:** Carolina Tripp-Barba, Aníbal Zaldívar-Colado, Gloria-María Peña-García, José-Alfonso Aguilar-Calderón, Ana-Rosa Medina-Gutiérrez

**Affiliations:** 1 Faculty of Informatics Mazatlan, Autonomous University of Sinaloa, Mazatlan 82117, Mexico; ctripp@uas.edu.mx (C.T.-B.); ja.aguilar@uas.edu.mx (J.-A.A.-C.); 2 Nursing School, Mazatlan, Autonomous University of Sinaloa, Mazatlan 82117, Mexico; gpena@uas.edu.mx (G.-M.P.-G.); anarosa_uas@hotmail.com (A.-R.M.-G.); 3 General Hospital Dr. Martiniano Carvajal, Mazatlan 82127, Mexico

**Keywords:** e-learning, COVID-19, computer science teaching, distance education

## Abstract

The COVID-19 pandemic has affected educational institutions around the world. One partial solution for students and teachers to continue the academic process involved the use of software and hardware technologies via the internet. The main objective of this research was to analyze the actions carried out by computer science teachers (and teachers who taught related degrees) in Sinaloa, Mexico, during the COVID-19 confinement period, to determine if the working conditions were different at all educational institutions. Based on quantitative, descriptive–explanatory, correlational, field, and cross-sectional approaches to data collection—a survey was designed and sent to teachers who taught classes in computer science and related careers. The results showed that although teachers felt prepared in designing and implementing virtual courses (90.73%), 68.5% believed that virtual classes were not enough for students (i.e., regarding replacing the training being offered). Likewise, teachers observed that only 27.8% of their students showed real commitment to online classes. In the hypothesis test, a chi-squared value of 3.84 was obtained, with a significance (*p*-value) of 0.137. There was a probability of error of 13.7%; this is high, considering that the level of significance must be 0.05 (5%) or less. It was concluded that teachers must be permanently trained in the use of new digital technologies; in addition, they must continuously produce academic material and make it available to the educational community. It is necessary for universities to design plans for the regulated use of applications and devices for academic purposes, update study plans and programs, and train teachers and students beyond conventional education.

## 1. Introduction

The World Health Organization (WHO) characterized COVID-19 as a pandemic after a wave of infections spread around the world. COVID-19 is an acute respiratory disease caused by the SARS-CoV-2 coronavirus. Governments around the world have responded to this pandemic by taking precautionary measures to stop its spread. Some of these measures include the closures of (all levels of) educational institutions. Millions of universities and schools have been closed as part of social distancing measures, thus limiting the spread of the virus. On 23 March 2020, UNICEF, based on UNESCO data, reported that, in Latin America and the Caribbean, around 154 million children (more than 95% of those in enrollment) were temporarily out of school—this period lasted for at least six months [1].

According to data from the Economic Commission for Latin America and the Caribbean (ECLAC), students from Latin America and the Caribbean already faced deteriorated social situations prior to the pandemic, due to issues such as poverty, inequality, or social discontent [2].

COVID-19 is the greatest challenge faced by national and international educational systems. Several governments have ordered institutions to suspend face-to-face classes for most of their students. Educational institutions face new challenges due to the pandemic. COVID-19 has influenced the way classes are taught; teachers and students stay at home and digital technologies provide support for studies to continue. Social media, video conferencing software, emails, and virtual learning environments, through desktop computers, laptops, tablets, and mobile phones, are now common tools in the teaching–learning process. The pandemic has not only impacted how we impart and receive knowledge, but it has also impacted the academic supply and demand. In this regard, internet bandwidth is relatively low with less access points, and internet services are costly in comparison to the average income in many developing countries, Mexico is no exception—accessibility is inadequate. Students without access to computers, mobile devices, or internet connections, for economic or geographical reasons, are deprived of access to courses; the same could happen with teachers. Affordability and accessibility for all students and teachers is a challenge that developers of educational tools should focus on. On the other hand, students who take classes online spend a greater amount of time connected to virtual platforms; this represents a potential problem because it leaves students vulnerable to online exploitation. The increasing (and unstructured) amount of time spent learning online has exposed students to dangerous and violent content, as well as an increased risk of cyberbullying.

Therefore, there could be a fall in the supply and demand of education, which could lead to a reduction in human capital, an increase in learning poverty (and poverty in general due to school dropouts), inequality, social unrest, as well as reinforcement of the intergenerational cycle of poverty and low human capital [3,4]. This would lead to a decline in the quality of education and affect the future of young people [3,5].

Currently, academic institutions have opportunities to improve the means and methods of the teaching—learning process through non-conventional and innovative educational modalities using technology, e.g., via applications and portable/mobile devices, which became necessary and popular during the pandemic, not only in academia but also among the general population. These technologies helped in the transition to e-learning in a short time and with relative success. The experiences gained should be used in the future.

Based on the above, the following research question arises—were the working conditions of teachers (in computer science and related degrees) in Sinaloa, Mexico, during the COVID-19 confinement period, different at all educational institutions? In this research, we analyzed the actions carried out by computer science teachers (and teachers with related degrees) in Sinaloa, Mexico, while teaching from home due to COVID-19 confinement. This was carried out under the assumption that teaching staff at the universities in Sinaloa, Mexico, were not prepared to teach from home via the internet, as they did not have the training or infrastructure, i.e., having relatively low internet bandwidth with less access points, costly internet services in comparison to income, or (lack of) equipment or skills necessary to carry out their work during the COVID-19 pandemic.

With the results of this research, we will know the needs and problems teachers faced during the pandemic period when they taught virtually. Problems were reflected in the teaching preparations and school infrastructures, which impacted student learning. It is necessary to understand the panorama of Sinaloa (in this matter) and offer a series of recommendations regarding the possible actions to be carried out to improve performances in case virtual classes continue. This investigation could serve as a starting point for substance reforms as well as in the plans and programs at universities in Sinaloa, Mexico. It highlights the reality of education in a health emergency, reflecting the needs of the teacher and student. This will allow them to face better possible contingencies that may arise in the future (due to a new pandemic or a resurgence of the current one). The theoretical support of this article could be relevant for the Ministry of Public Education in Sinaloa, by providing strategic axes to follow, to strengthen the education in the face of possible health contingencies. It could even serve as a topic of debate for higher education experts regarding the definition of reforms and their applicability in the prevention of health problems.

The article is organized into five sections. Section 2 presents the state-of-the-art studies related to this research; Section 3 explains the methodology carried out. The results and discussion are presented in Section 4 and Section 5, respectively. Finally, the conclusions are presented in Section 6.

## 2. State-of-the-Art

COVID-19 has led institutions to modify their classroom teaching strategies (to virtual classrooms). There are many studies on this topic. For example, ref. [6] talked about new relationships between society, students, and teachers. This study discussed the new challenges, i.e., the planning, development, and evaluation of classes being conducted from the home and the need to generate conducive environments and a greater interest in training. Technology allows us to generate digital documents and audiovisual materials and it ‘accompanies’ us in developing classes.

Britez [7] addressed how educational measures were taken in countries such as Paraguay, Argentina, and Brazil since the first positive case of COVID-19 was discovered. It should be noted that, globally, virtual classes took place in order for the school year to continue; digital platforms were enabled and various technological resources and applications (e.g., WhatsApp and Classroom) were used. It was possible to identify problems where teachers and parents were unprepared for virtual environments; situations where it was not possible to carry out activities virtually were highlighted. In the same idea, but focusing only on Argentina, ref. [8] studied technological indicators and how teachers used these resources in distance education during the pandemic. WhatsApp was mentioned as the most-used medium, even allowing disadvantaged teachers and students to continue with the educational process. According to the teachers, this situation had a negative influence on student performances. In [9], the authors looked into the descriptions of the role changes that teachers and students faced when resorting to virtual learning, via forums such as wikis, meetings, Zoom, Skype, Classroom, Moodle, etc. The authors stated that students must work in collaboration with others to achieve the objectives and force teachers to use computer tools (for the development of digital educational content). The study concluded that this new work model has fostered certain ways of thinking and acting in the face of specific problems and that the use of the inverted class model is positive.

In [10], the authors discussed how the change from face-to-face to virtual education has mainly affected students from the most disadvantaged socioeconomic strata; this was seen in an analysis carried out in Ecuador, but could easily be the case in many Latin American countries. In this study, it was mentioned that 75% of households in Ecuador do not have computers to carry out virtual education activities; in many cases, the time on a device must be divided between several children and the parent who is conducting remote work. Likewise, only 37.2% of households have an internet connection, which is why only 6 out of 10 children could continue with their education. In [11], the authors studied the main difficulties encountered by educational institutions and some strategies used in the teaching–learning processes, focusing on Latin America (particularly Peru, Mexico, Ecuador, Costa Rica, and Chile). Some of the main problems faced by teachers are the lack of resources and technological platforms and ignorance of pedagogical models. In the contingency plan that they exposed, they indicated aspects, such as educational level (basic, secondary, university, and special education), location (rural or urban), and attitude towards the use of technologies (of teachers, students, and administrative). Lloyd [12] focused on educational inequalities in Mexico; according to surveys, only 45% of Mexicans have computers and only 4% of the people in rural areas have access to the internet. Regarding teachers, a strong difference was also mentioned between public and private schools; it is expected that those who work in private schools have greater access to online technologies and, therefore, their students have better opportunities to take advantage of resources. It was concluded that it is critical to find solutions that close the technological gaps in the country.

The authors of [13] looked at the comparison between the teaching–learning processes adopted between Mexico and Argentina due to the virtual learning impact; the measures adopted by universities in said countries were analyzed. Students and teachers participated in this research, concluding that in contrast to Argentina (where they were adapted to the use of virtual environments as well as less transportation costs due to virtual learning), in Mexico, not having equipment (computer or a laptop) and a lack of (or a bad) internet connection, was the norm. It was concluded that, as part of the new teaching process, methods should be sought to motivate, guide, and establish student concentration, and above all to find a way to uncover more participatory roles.

With online teaching being forced into effect by this exceptional problem worldwide—universities have never faced massive online assessments before (from an institutional perspective). Therefore, teachers and students have had to collaborate to provide responses that integrate methodological and technological decisions while guaranteeing equity, legal security, and transparency for all actors, internally and externally. This was presented by [14], who presented the recommendations made by the Group of Online Teaching Managers of the Public Universities of Castilla y León; recommendations are aimed at designing online evaluation mechanisms and strategies, leading to evaluation processes that are fair for everyone.

In [15], the author wrote about primary and secondary school children; after just 20 days of confinement in Spain, it was the sector most affected by COVID-19 restrictions. Above all, his study focused on identifying the problems that this confinement will have when life returns to normal since it identifies problems that the children were involved in during this time, including feelings of loneliness, domestic violence, family economic situation, etc., and how they should try to cope once the confinement passes. Hall [16] analyzed the serious problem that Mexico faces regarding sedentary lifestyles and childhood obesity (which will increase due to the use of virtual education) as well as the sedentary levels of primary school students during the pandemic.

We should note the analysis made about the perception of learning in a virtual environment and the use of ICT, developed at the Universidad Autónoma de Nuevo León [17] and the Universidad Autónoma de Chiapas [18]. Moreover, the study highlights how public universities faced while moving into distant teaching practices at Universidad Autónoma Metropolitana [19] and Yucatán, México [20]. Furthermore, teaching challenges (regarding the digitalization process in German schools) were presented by [21,22]; the authors presented a study on the higher education teaching practices experienced in Mexico due to the switch from face-to-face teaching to emergency remote teaching. The analysis by [23] concluded that humans learned more about technology and education in 2020 than in the previous ten years; it was also noted that during the COVID-19 confinement, the ’mastering’ of app and device usage improved considerably despite the time demands of continuously developing new material in classes. In [24], the authors analyzed how primary and secondary schools in Catalonia (Spain) implemented the teaching and learning process during the lockdown, showing a ‘digital range’ involving students and teachers. They concluded that teachers from private institutions generally presented better conditions for transitioning to the new model without further setbacks. Similarly, their results indicated a lack of teachers’ familiarity with various digital tools, i.e., to facilitate their remote teaching experiences. Finally, the authors of [25] studied different educational models caused by the COVID-19 pandemic: face-to-face and e-learning. The main conclusion was that the students preferred to continue with the face-to-face learning process (49%) rather than online learning (7%) or, failing that, mixed or blended learning (44%), where the theoretical classes could be online and the practical classes could be face-to-face.

As shown in this section, there is a considerable amount of research on how the COVID-19 health emergency has impacted education. For years, there has been talk of strategies for distance education, for example, in the work carried out by [26], where the difficulties faced by this educational modality were discussed; the quality of courses and terminal efficiency could be improved by detecting improvement strategies and areas of opportunity.

Various activities had to be modified due to the pandemic, specifically in the educational environment, and teachers and students were immersed in new ways of working; this included students at all educational levels (from primary to postgraduate) and various activities (classes, practices, social services, tutorials, etc.).

## 3. Methodology

A quantitative approach was used at a descriptive–explanatory level. This was a field study, because first-hand data were obtained directly from primary sources—the teachers. It was cross-sectional; the measurements were made in single moments.

The Mexican educational system is divided into basic, middle, and higher levels. The basic level is made up of preschool (from 3 to 6 years old), primary (from 6 to 12 years old), and secondary education (from 12 to 15 years old); the middle level is high school (from 15 to 18 years old); the higher levels include bachelor’s, masters, and doctorate degrees. The study population was made up of higher-education professors from Sinaloa, Mexico, who work in technology-related programs, with bachelor’s degrees in computer science, animation and visual effects, information systems engineering, mechatronics, biotechnology, and software, among others. To obtain data, the authors designed a structured survey with 30 items divided into six categories—(i) demographic information; (ii) labor information; (iii) infrastructure and teacher training; (iv) teachers’ opinions on the use of digital technology by the student; (v) teaching skills for virtual teaching; (vi) effects of COVID-19 in education; the surveys were self-completed by the teachers, guaranteeing anonymity.

In the survey, teachers were questioned about their demographic and employment information (gender, age, workplace, area, and highest degree of studies). Opinions were collected on the infrastructure offered by the institutions where they worked, and whether they had tools such as a computer, the internet, or smartphones in their homes. Likewise, they were asked if they had recently received training in relation to ICT or the development of virtual educational activities. The ‘instrument’, in the same way, covered their opinions in relation to the use of digital technology by the students, if the teachers felt that they had the necessary knowledge to design and teach online courses, and finally, their perceptions about students accepting the new way of receiving classes. The survey was implemented on the web via Google Forms and distributed by email and social media (WhatsApp and Facebook) to the participants. In total, 540 higher education teachers responded from Sinaloa. We protected the rights of the research participants, enhanced the research validity, and maintained scientific integrity [27]. Moreover, the ethical considerations contained in the Regulations of the General Health Law in the field of Research for Health [28] were taken into account, specifically the second title of the Ethical Aspects of Research in Human Beings, Chapter I, articles 13, 16, and 23.

The answers provided by the teachers are divided into categories: (i) demographic information, (ii) labor information, (iii) infrastructure and teacher training, (iv) opinion on the use of digital technology by the student, (v) teaching skills for virtual teaching, (vi) effects of COVID-19 on education. The responses to the items in these categories are Likert-type: 5—totally agree, 4—agree, 3—neutral, 2—disagree, 1—totally disagree; and dichotomous: 2—yes, 1—no. As can be seen, the instrument was designed so that the positive responses had higher scores; when adding the scores obtained by each study subject, those with higher numbers were considered as having better training, infrastructure, skills, and perceived better conditions in their students. This summation was carried out in the scores to determine if the conditions in the three institutions were homogeneous.

Correlational analyses were carried out by the institutions, but only with two universities, UAS and UPSIN, as only 10 subjects from the UdeO responded to the survey. Therefore, a Pearson correlation coefficient greater than 0.6, obtained between all variables, was compared with the data of the two institutions individually, not between them, with the objective to make a comparison, finding similarities and differences.

The analysis was carried out with 30 variables, 29 of them independent and 1 dependent: the institution where the teacher worked. To determine the distribution of the data, the Kolmogorov–Smirnov test was performed with the Lilliefors significance correction, since *N* = 540. Subsequently, the chi-squared value of homogeneity was calculated to evaluate the research hypothesis.

Table 1 shows the instrument’s internal consistency, which obtained a Cronbach’s alpha of 0.57. Thus, it was considered reliable and acceptable. According to the dimension or section (Dichotomous), a score of 0.88 was obtained according to Cronbach’s alpha statistic; the Likert-type section obtained a score of 0.99, making it reliable and acceptable. The dimensions or sections reached reliability of 0.98.

## 4. Results

In this section, the results obtained from the survey are analyzed and interpreted. The data collected from each of the six categories addressed are presented and used to study the information of the teachers participating in the survey.

Table 2 shows the gender and age distribution of the teachers who responded to the instrument. These data correspond to the first category of the survey “Demographic information”, where information, e.g., gender, age, and city where they worked, area (urban or rural), and the highest degree of study was requested.

As can be seen in Table 2, the last column and last row show the total figures in each category. It can be observed that the number of male teachers was higher than females, at 310 (57.40%) and 230 (42.59%), respectively. The ages ranged from 30 to 65, with a higher frequency of those from 35 to 39.

Regarding the maximum degree of study, while using the instrument to obtain data, four options were considered: bachelor’s degree, specialty, master’s degree, and doctorate. The responses are organized by gender and institution in Table 3.

According to the summary observed in Table 3, 220 (40.74%) professors claimed to have a master’s degree, 210 (38.88%) had a doctorate, 100 (18.51%) had a bachelor’s degree, and 10 (1.85%) had a specialty degree. All respondents worked in urban areas, at the Universidad Autónoma de Sinaloa (UAS), Universidad Politécnica de Sinaloa (UPSIN), and the Universidad Autónoma de Occidente (UAdO); this was uncovered within the second category “Labor information”, where they were asked to indicate where they worked.

In the third category, “Infrastructure and teacher training”, in the statements about whether their institutions provided them with computer equipment and an internet connection to teach their in-person classes, 57% of teachers mentioned that the institutions where they worked did not provide a computer for use during the school day, and only 41% had an internet connection; 530 (98.14%), 540 (100%), and 500 (92.59%) answered affirmatively to each of the statements about whether they had the necessary tools at home, such as a computer, internet connection, and a smartphone, respectively. Finally, 35 and 48% said they had not received any training regarding ICT or virtual teaching in the last three years, respectively.

The fourth category, “Teaching opinion on the use of digital technology by the student”, includes statements about whether the students had the necessary tools at home, such as a computer, internet connection, and smartphone; the information obtained is presented in Table 4. According to 300 teachers (55.5%), between 81 and 100% of their students had a computer at home. In the case of smartphones, 310 teachers (57.4%) answered that 81–100% of their students had one, but only 220 teachers (40.74%) answered that between 81 and 100% of their students had an internet connection; therefore, it would be impossible for them to continue with classes remotely.

As can be seen in Table 4, only 55.5% of the students had computer equipment and 57.4% had a smartphone; this allowed us to identify the deficiencies in terms of preparing students to receive virtual classes. This was mainly due to the lack of financial resources. Finally, it can be seen that less than half of the students had an internet connection; that is, only 40.74%. This indicates that even when some of the students had some equipment to work with (laptop, desktop computer, smartphone) it could be the case that they did not have an internet connection, which further reduced the number of students who were prepared to continue with online classes through virtual platforms and video calls, meaning that, even though the students had the means to adapt to the new teaching modalities, it was impossible for them to attend classes, have the proper material, and be able to carry out tasks and activities on time.

The next category of the survey, “Teaching skills for virtual teaching”, included questions related to the teachers’ perception of whether they had the necessary knowledge to teach their courses virtually. Table 5 summarizes the items in this section, which are Likert-type and on a scale ranging from 1 to 5—1 for “Totally disagree”, 2 “Disagree”, 3 “Neither agree nor disagree (neutral)”, 4 “Agree”, and 5 “Totally agree”.

As illustrated in Table 5, the statements “I have the knowledge to design virtual courses”, “I have the knowledge to implement courses on a virtual platform”, “I have taught online courses through virtual platforms”, “I would like to teach courses creating educational videos in addition to the platform”, “I have already created and used videos to support my face-to-face or online courses”, and “I teach subjects that are mainly practical” received the higher answers, with values between 53.70% and 66.66% for “Strongly agree”. Mostly, teachers (220) were neutral regarding whether “A video tutorial on techniques and procedures is enough for the student to reinforce their theoretical knowledge”, which had 40.74%. Even when the professors said they had the knowledge to create educational content, 210 (38.88%) and 160 (29.62%) answered “Strongly disagree” and “Disagree”, respectively, to “A video tutorial on techniques and procedures is enough for the student to reinforce their theoretical knowledge”. Finally, to the item “My students acquire the same skills by receiving online and face-to-face classes”, the teachers mostly responded with “Strongly disagree” and “Disagree” (31.48% and 24.07%).

Table 6 presents the questions from the category “Effects of COVID-19 on education”, which focused on how teachers perceived the experiences/learning of students via this type of education. These questions, similar to the previous category, are Likert-type on a scale that ranges from 1 to 5—1 for “Strongly disagree”, 2 “Disagree”, 3 “Neither agree nor disagree (neutral)”, 4 “Agree”, and 5 “Strongly agree”.

As can be seen in Table 6, before the statement “I feel comfortable using ICT to teach my classes”, 360 (66.66%) of the teachers said they “Strongly agree”, but they did not notice if the students were engaged or had positive attitudes working remotely; moreover, they did not perceive any learning improvements in their students while working at a distance. As for negative issues during the COVID-19 period, it was identified that the institutions did not have the necessary infrastructure to teach face-to-face classes without risking the health of students and teachers due to COVID-19. Thus, professors believed that they would be put at risk if they returned to teach face-to-face classes.

Table 7 shows the Pearson correlation coefficient calculated individually by institution (only from the UAS and UPSIN). This analysis was not performed with the data from the third university, UdeO, as only ten subjects answered the survey.

As seen in Table 7, there was a rise in the variables “Percentage of students who have a computer at home” and “Percentage of students who have Internet access at home” (0.79 in the UAS and 0.89 in the UPSIN). Between the variables “I have the knowledge to design virtual courses” and “I have the knowledge to implement courses on a virtual platform”, the correlation was 0.94 in the UAS and 0.80 in the UPSIN. Moreover, among the variables “Online education is sufficient for the student and can replace laboratory work or practical training”, and “My students acquire the same skills by taking online and face-to-face classes” similarities were found in the correlation, resulting in 0.80 in the UAS and 0.73 in the UPSIN. Finally, there was a correlation of 0.60 in the UAS and 0.67 in the UPSIN, for the variables “I perceive an improvement in the learning of my students when working at a distance”, and “My students have the skills to learn online”. The rest of the results show a correlation <60 for some of the institutions and >60 for others.

To obtain the distribution of the dependent variable, made up of the sum of the data from categories (ii) labor information, (iii) infrastructure and teacher training, (iv) teaching opinions on the use of digital technology by the student, (v) teaching skills for virtual teaching, and (vi) effects of COVID-19 on education—the Kolmogorov–Smirnov test (Table 8) was performed with the Lilliefors significance correction, since the sample was composed of 540 data points.

**Figure 1:**
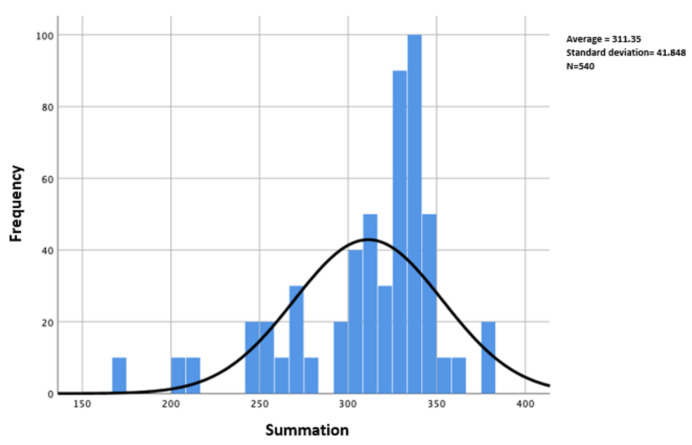
Histogram of the data distribution of the dependent variable (teacher’s institution).

As can be seen in Table 8, the *p*-value is almost zero ($7.075\times 10^{-50}$), so the distribution is not normal. This is confirmed in Figure 1. The analysis of the data must be carried out with non-parametric tests because it presents this type of distribution,

Table 9 shows the frequency distribution of teachers with scores higher and lower than 300, by institution.

Table 9 shows the number of teachers and their percentages by institution (UAS, UPSIN, and UAdeO), with scores higher and lower than 300.

To answer the hypothesis—the human, infrastructure, and training conditions necessary to carry out the teaching work and implement the expected learning were different at each university in Sinaloa, Mexico, during the COVID-19 pandemic. The chi-squared value of homogeneity was calculated; it was 3.977 (Table 10). The significance (*p*-value) was 0.137 (13.7%).

## 5. Discussion

The SARS-CoV-2 coronavirus pandemic has caused profound changes in society, which have not been gradual, but immediate; these changes surprised the world as, globally, we lacked the necessary preparations. Many activities are now carried out from home (online). Universities and their different actors—students, teachers, managers—are not immune to this disaster, but based on the experiences from overcoming other epidemics, the importance of continuing with daily activities, as much as possible, has been demonstrated. As stated by the Centers for Disease Control and Prevention (CDC), “in a pandemic […], these [academic] institutions must maintain a balance between academic continuity, infection control, and minimization of morbidity” [29].

To alleviate infections and reduce the pandemic’s impact, as well as avoid student and teacher absenteeism, government authorities (globally), including in Mexico, opted for online education, relying on internet technology and the services it offers. Lifestyle changes, which imply modifying the habits and behaviors of the population, were needed. In addition to fostering isolation between society, including students and teachers, this new way of living also assumes that the educational process continues, whether it be with the help of social media, email, or learning platforms. This is conducted with the goal to reduce infections—until the outbreak disappears or while an effective vaccine is developed.

Therefore, the teachers participating in the study were questioned as to whether whether a video tutorial (on techniques and procedures) would be enough for the students to ‘reinforce’ their theoretical knowledge—only 90 said they fully agreed or agreed, 220 did not agree or they disagreed, and 140 disagreed or strongly disagreed. This contrasts with the findings by [30,31], who affirmed that these instruments favor the involvement of students in the educational process, helping them to strengthen their knowledge and narrow the learning gaps, turning video into an effective educational tool.

Only 90 professors claimed to fully agree or agree that online education was sufficient for the student to replace laboratory or practical training; the rest maintained neutral positions or disagreed. This perception is different from the conclusion reached by [32]: online education in some institutions has increased the access to information by teachers and students. A rich, collaborative environment for students can improve academic standards. It also differs from the results by [33], who affirm that the use of e-learning is positively and significantly related to student satisfaction, having an impact on the intention of use that, in turn, affects the effectiveness of e-learning.

The previous opinion disagrees with the assessment of the students—300 teachers said that they acquired the same skills when taking classes online as in person (56%); the other 44% (240 teachers) were neutral or were in disagreement. These results coincide with the research by [34], who carried out a study using a blended learning model with a hands-on approach for in-service secondary school teachers. They looked at the combination of e-learning and face-to-face discussions, finding that access, flexibility, cost-effectiveness, improved interaction, forming a teacher network, and the participation of administrators, instructors, and school leaders were factors that contributed to the success of the learning model. This coincides with the findings by [35], who found that the use of e-learning […] for medical students was an interesting alternative and an effective method to develop competence.

Only 150 teachers considered that their students were engaged and had positive attitudes toward working remotely. The rest, 390 teachers, ranged from having neutral positions to completely disagreeing. The previous assertion does not coincide with the research by [36], who found that e-learning in higher education could have a positive impact on the learning process, such as greater satisfaction, motivation, and greater student engagement. It also did not coincide with the research by [37], who provided evidence about the achievement of the reflective commitments of students […]. The designed initiative allowed students to achieve significant gains in knowledge to improve their understanding of e-learning. Students perceived that the initiative could help them achieve their learning outcomes. They also perceived that group interactions and the exchange of experiences with peers, teachers, and related experts in this context could help advance their knowledge.

Given the statement “I perceive an improvement in the learning of my students when working at a distance”, 150 teachers (28%) completely agreed or agreed with it; however, the vast majority, 390 teachers (72%), ranged from neutral positions—through disagreeing to completely disagreeing. These results coincided with those by [38], who showed that learning performance was higher in a flipped classroom environment compared to other learning environments, such as traditional, e-learning, and b-learning. Students in the b-learning environment had better learning performances compared to the e-learning environment.

However, the results differed from a study on the implementation of m-learning in education, where it was found that mobile adaptations compared to e-learning approaches had limited impacts (on the performances) in learning practical skills. Information was also collected on the use of mobile systems (which were developed by the authors according to the contents of the classes taught, with mobile and web interfaces) and was compared with traditional computer access. The results suggest that students learned in a similar context regardless of the way they accessed learning content. This may lead to the questioning of current assumptions on student mobility [39].

## 6. Conclusions

Implementation (planned or not) of online learning must occur with appropriate strategies. Students must have the skills required by this challenge; trained teachers, institutional support, and the necessary infrastructure for teachers and students must be available. There is a significant relationship between e-learning strategies and the increase in the efficiency of educational performance in universities [40].

Due to the statistical results, the hypothesis cannot be accepted because there is not enough evidence to conclude that the variables are associated. So, the following hypothesis is rejected: the human conditions, infrastructure, and training necessary to carry out teaching work and achieve the expected learning were different at each university in Sinaloa, Mexico, during the COVID-19 pandemic. Therefore, it is concluded that, in the universities in Sinaloa, Mexico, there were no significant differences in human resources, infrastructure, and training regarding the implementation of online education during the COVID-19 pandemic. Additionally, it was observed (Table 8) that the teachers of UAdeO presented less favorable conditions (66.7%) than those of UAS (71.4%) and UPSIN (78.3%).

The results of this study will be valuable in case of another contingency, whether natural or artificial. The main recommendations could be divided into three sections: teachers, students, and government or educational authorities.

Regarding teachers—they must be permanently trained in the use of new digital technologies, which are constantly evolving; in addition, they must constantly produce academic material and make it available to the educational community. Therefore, the docents should be prepared to use virtual platforms, such as Moodle, Google Classroom, Microsoft Teams, etc. Moreover, they need to be prepared to elaborate on class material via the use of videos or support lecture documents that back up the information in class (in case the student cannot be present physically). This should be included in the preparation of all teachers, particularly in the technological areas.

For teachers, it is essential to accept that a more personalized and agile approach is needed for this type of education, for example, more personalized communication with students throughout the semester and the provision of tailor-made solutions that meet the needs of students. Furthermore, regarding evaluations, it is necessary to design more appropriate strategies and synchronize them with how it is taught. In this sense, methods, such as creating activities in mobile applications (e.g., Kahoot!), writing blogs, publishing videos on YouTube, generating mental maps and preparing presentations in different interactive formats, should be considered.

Students should receive instructions on how to use ICT (this knowledge should be updated as often as necessary). The essential issue is to offer students virtual environments and prepare extra courses, seminars, or video tutorials about the general use of the tools. It is difficult for a student to follow the class in an environment that is complicated to manage or one that the student does not understand.

A positive result of this situation (virtual learning due to the pandemic) involves the support provided by international corporations, such as Microsoft and Google. The former involves Teams; the latter involves Google Classroom and Google Meet. Both provide free online courses and certifications for teachers and have made alliances with universities for free licenses.

Finally, administrators of educational institutions, governments, and educational authorities at all levels must seek financing options, both internally and externally, to keep their IT infrastructures as updated as possible, and sufficient for teachers and students. Regarding lower-resource students, spaces that provide continued education should be offered, ensuring that all students have access to the minimum hardware and software, as well as high-bandwidth internet connections, to continue their learning in situations similar to the one that has prevailed in the world at present.

This study has limitations. The sample was restricted to teachers who teach undergraduate courses in the areas of computer science at three public universities in the southern Mexican state of Sinaloa. Obtaining data in electronic form may have excluded people without internet connections. The application of the survey during the COVID-19 pandemic period may not have resulted in accurate answers due to other obvious concerns in the population and the possibility that some teachers had symptoms of the disease.

## Figures and Tables

**Table 1:** Internal consistency of the survey.

Survey	Item	Total	Alpha
Questions	15, 16, 17, 18, 19, 20, 21, 22, 23, 24, 25, 26, 27, 28, 29 and 30	16	0.990
Computer skills questions	6, 7, 8, 9, 10 and 11	6	0.880
All questions, including percentages (except gender, age, city, or state where you work, highest degree of study, and institution where you have your highest load)	6–30	25	0.579
Dichotomous questions and Likert-type response questions	–	22	0.984

**Table 2:** Age and gender distribution.

Age	Male	Female	Total
30–34	20	50	**70**
35–39	110	40	150
40–44	50	40	90
45–49	60	40	100
50–54	30	40	70
55–59	20	10	30
60–65	20	10	30
Total	310	230	540
	(57.40%)	(42.60%)	

Source: Original, developed during the investigation.

**Table 3:** Degree of study by institution and gender.

Institution	Bachelor’s Degree		Specialty		Master’s Degree		Doctorate		Total
Universidad Autónoma de Sinaloa	0	0	0	10	60	50	120	60	300
Universidad Politécnica de Sinaloa	50	50	0	0	50	50	10	20	230
Universidad Autónoma de Occidente	0	0	0	0	10	0	0	0	10
Total	50	50	0	10	120	100	130	80	
	9.25%	9.25%	0	1.85%	22.22%	18.51%	25%	14.9%	

Source: Original, developed during the investigation.

**Table 4:** Teachers’ opinions on the use of digital technology (by the students).

Statement	0–20%	21–40%	41–60%	61–80%	81–100%
Percentage of students who have a computer at home	0	20 (3.70%)	60 (11.11%)	160 (29.62%)	300 (55.55%)
Percentage of students who have a smartphone at home	0	10 (1.85%)	80 (14.81%)	140 (25.92%)	310 (57.40%)
Percentage of students who have internet access at home	20 (3.70%)	0	70 (12.96%)	230 (42.59%)	220 (40.74%)

Source: Original, developed during the investigation.

**Table 5:** Teaching skills for virtual teaching.

Statement	1	2	3	4	5
I have the knowledge to design virtual courses	10 (1.85%)	0	40 (7.40%)	170 (31.48%)	320 (59.25%)
I have the knowledge to implement courses on a virtual platform	10 (1.85%)	0	40 (7.40%)	180 (33.33%)	310 (57.40%)
I have taught online courses through virtual platforms	20 (3.70%)	50 (9.25%)	20 (3.70%)	130 (24.07%)	320 (59.25%)
I would like to teach courses creating educational videos in addition to the platform	10 (1.85%)	30 (5.55%)	60 (11.11%)	80 (14.81%)	360 (66.66%)
I have already created and used videos to support my face-to-face or online courses	30 (5.55%)	80 (14.81%)	70 (12.95%)	70 (12.95%)	290 (53.70%)
I teach subjects that are mainly practical	10 (1.85%)	30 (5.55%)	80 (14.81%)	70 (12.95%)	350 (64.81%)
A video tutorial on techniques and procedures is enough for the student to reinforce their theoretical knowledge	50 (9.25%)	40 (7.40%)	220 (40.74%)	130 (24.07%)	100 (18.51%)
Online education is sufficient for the student and can replace laboratory work or practical training.	210 (38.88%)	160 (29.62%)	90 (16.66%)	60 (11.11%)	20 (3.70%)
My students acquire the same skills by taking online and face-to-face classes	170 (31.48%)	130 (24.07%)	110 (20.37%)	100 (18.51%)	30 (5.55%)

1: “Strongly disagree”, 2: “Disagree”, 3: “Neither agree nor disagree (neutral)”, 4: “Agree”, and 5: “Strongly agree”.

**Table 6:** Effects of COVID-19 on education.

Statement	1	2	3	4	5
I feel comfortable using ICT to teach my classes	0	10 (1.85%)	60 (11.11%)	110 (20.37%)	360 (66.66%)
I see my students engaged and with a positive attitude working remotely	40 (7.40%)	110 (20.37%)	210 (38.88%)	140 (25.92%)	40 (7.40%)
I perceive an improvement in the learning of my students when working at a distance	60 (11.11%)	190 (35.18%)	220 (40.74%)	50 (9.25%)	20 (3.70%)
My institution has the necessary infrastructure to teach face-to-face classes without risking the health of students and teachers due to COVID-19	160 (29.62%)	160 (29.62%)	120 (22.22%)	50 (9.25%)	50 (9.25%)
My health would be at risk, due to COVID-19, if I return to teach face-to-face classes	30 (5.55%)	20 (3.70%)	30 (5.55%)	60 (11.11%)	400 (74.07%)

1: “Strongly disagree”, 2: “Disagree”, 3: “Neither agree nor disagree (neutral)”, 4: “Agree”, and 5: “Strongly agree”.

**Table 7:** Pearson correlation coefficient calculated by institution.

Survey Items/Correlated Variables	UAS	UPSIN
	Pearson (r)	
**Percentage of students who have a computer at home**		
Percentage of students who have internet access at home	0.79	0.89
Percentage of students who have a smartphone at home	0.09	0.69
**Percentage of students who have internet access at home**		
Percentage of students who have internet access at home	0.22	0.74
**I have the knowledge to design virtual courses**		
I have the knowledge to implement courses on a virtual platform	0.94	0.80
**I have the knowledge to implement courses on a virtual platform**		
My institution has the necessary infrastructure to teach face-to-face classes without risking the health of students and teachers due to COVID-19	−0.17	−0.64
**I would like to teach courses creating educational videos in addition to the platform**		
I have already created and used videos to support my face-to-face or online courses	0.37	0.61
I would like to receive training in virtual teaching	0.11	0.72
**A video tutorial on techniques and procedures is enough for the student to reinforce their theoretical knowledge**		
Online education is sufficient for the student and can replace laboratory work or practical training	0.29	0.66
**Online education is sufficient for the student and can replace laboratory work or practical training**		
My students acquire the same skills by taking online and face-to-face classes	0.80	0.73
I perceive an improvement in the learning of my students when working at a distance	0.76	0.20
**My students acquire the same skills by taking online and face-to-face classes**		
I perceive an improvement in the learning of my students when working at a distance	0.73	0.33
**I see my students engaged and with a positive attitude working remotely**		
I perceive an improvement in the learning of my students when working at a distance	0.80	0.73
My students have the skills to learn online	0.20	0.62
**I perceive an improvement in the learning of my students when working at a distance**		
My students have the skills to learn online	0.60	0.67
My institution has the necessary infrastructure to teach face-to-face classes without risking the health of students and teachers due to COVID-19	0.65	−0.26

Source: Original, developed during the investigation.

**Table 8:** Kolmogorov—Smirnov test.

		Summation
*N*		540
Normal parameters	Average	311.35
	Deviation	41.848
Maximum extreme differences	Absolute	0.183
	Positive	0.121
	Negative	−0.183
Test statistic		0.183
*p*-value		$7.075\times 10^{-50}$

**Table 9:** Frequency distribution by institution.

Institutions								
	**UAS**		**UPSIN**		**UAdeO**		**Total**	
Summation	*N*	%	*N*	%	*N*	%	*N*	%
<300	80	28.6	50	21.7	10	33.3	140	25.9
>300	200	71.4	180	78.3	20	66.7	400	74.1
Total	280	100	230	100	30	100	540	100

**Table 10:** Chi-squared tests of homogeneity.

	Value	df	Significance (*p*-Value)
Pearson’s chi-squared	3.977	2	0.137
Likelihood ratio	3.922	2	0.136
N of valid cases	540		

## Data Availability

The data analyzed and obtained for this study are available at https://doi.org/10.6084/m9.figshare.20075897.v1 (accessed on 9 May 2022).
